# Bibliography

**Published:** 1901-12-15

**Authors:** 


### BIBLIOGRAPHY.

"INTERNAL ANATOMY OF THE FACE," by M. H. Cryer, M.D., D.D.S.—The S. S. White Dental Manufacturing Company has just issued in book form the work which Dr. Cryer has in large part contributed to dental societies and dental and medical periodicals during the last two years. We feel confident that no more important work has been done in recent years, and it is gratifying to have it gathered into a single volume where it is accessible and may be used in class work. It is a work of 175 pages, and is beautifully printed on heavy, highly-finished paper, so that the illustrations, some 160, show up as clearly as the original negatives. It is one of those cases where the printer has helped rather than maligned the author. The great value of this work lies in its originality and reliability. It is neither bookish, ideal or diagramatic, but photographs of sections cut directly from the cadaver or skull, with all the parts shown in their proper relations. It will not, and is not intended to, supplant the more extensive text-books on anatomy, but will serve to correct, or at least modify, many of the dogmatic statements contained in our text-books. The text of the book is not the least valuable part of the work. While in the main it is intended to explain the illustrations and call attention to digressions from the normal or supposed conditions, it is so clearly and concisely written as to not only make a remarkably comprehensive statement of the anatomical essentials, but furnish so much that is new and fresh as to make it exceedingly interesting and attractive reading. It is a book that should be in the library of every dentist.

"Gray's Anatomy."—A new edition, the fifteenth in number, has just come from the press of Lea Bros. & Co. We have not read it through, but we have been sufficiently curious to turn its pages one by one, more than twelve hundred of them, and compare it mentally with the copy we read with so much care more than twenty-five years ago. We note some of the old landmarks in the cuts, and the text in most places has a familiar appearance. But there are so many changes in places that it really seems like another book. Many new cuts are added and others seem to have been redrawn. Every part seems to have been elaborated, and whole chapters of new material added. Many of the cuts are made intelligible by the use of colors, which enables one to fix the facts at a glance, a very helpful scheme. Whilst so many additions and changes are noted, we are impressed with the fact that the general plan of the work remains unchanged, and each part of it has been brought into harmony with the results of the latest research and given such space and attention as its merit deserves. With all this wealth of new material and illustration the work has been kept well within the bounds of a text-book. This fact and the ideal conception of its original author, Henry Gray, have made Gray's Anatomy the standard of all anatomical text-books; and, so long as its revisers and publishers keep this plan as a basis, this work will continue to meet the needs of teachers and students of anatomy. The sections of special interest to dentists have received proper attention of the revisers, and we are glad to note that Dr. Cryer's work on the bones of the face has been given recognition. It is remarkable in this connection that so expensively made a book can sell for so small a price—$5.50.

The fourth edition of Talbot's "IRREGULARITIES OF THE TEETH" has just come from the press of the F. A. Davis Co., of Philadelphia. The work is greatly enlarged from the former edition. This work differs essentially from all others on this subject, in that it deals almost entirely with theories which pretend to account for the various malformations of the jaws and teeth, and the relative positions which they occupy in malformations of similar kinds and which the author attempts to account for and classify. While it may seem that the work lacks technical or applied mechanics for the correction of irregularities, the author maintains that it is essential that the cause be known, whether of local or systemic origin, before any intelligent use may be made of the mere mechanical efforts to correct malpositions of the teeth in the arch. He does not advocate any peculiar system of method or appliance for regulating teeth, except to illustrate a few cases which have been successfully treated in his practice by appliances of his own devising. The work shows an immense amount of careful study and research and is an invaluable contribution to the literature of this subject. The studious specialist will find much in it to interest and edify; but the general practitioner will want more citations of cases and methods used in their correction. The profession is under great obligation to Dr. Talbot for his devotion to this aspect of one of the most important subjects of dental practice, and particularly for the placing of the results of his labors in such accessible form. We trust the book may have a large sale and a careful reading.

